# Lower Prevalence of Disordered Eating Behaviours Among Norwegian Female Athletes Compared to Non‐Athletes: A Cross‐Sectional Survey Using the Eating Disorder Examination Questionnaire

**DOI:** 10.1002/ejsc.70043

**Published:** 2025-08-20

**Authors:** Mimmi M. S. Vedenpää, Edvard H. Sagelv, Monica Klungland Torstveit, Kristin Benjaminsen Borch, John Owen Osborne

**Affiliations:** ^1^ Department of Community Medicine Faculty of Health Sciences UiT The Arctic University of Norway Tromsø Norway; ^2^ School of Sport Sciences Faculty of Health Sciences UiT The Arctic University of Norway Tromsø Norway; ^3^ Department of Sport Science and Physical Education University of Agder Kristiansand Norway; ^4^ Sunshine Coast Hospital and Health Service Birtinya Australia; ^5^ School of Health University of the Sunshine Coast Sippy Downs Australia; ^6^ School of Exercise and Nutrition Sciences Queensland University of Technology Brisbane Australia

**Keywords:** eating behaviours, eating disorders, EDE‐Q, leanness, women

## Abstract

The purpose of this study was to report and compare the prevalence of disordered eating behaviours (DEBs) among Norwegian female athletes of different competition levels and sport types, and non‐athletes of different physical activity levels. A total of 565 females (athletes: *n* = 189; non‐athletes: *n* = 376) completed the Eating Disorder Examination Questionnaire 6.0 (EDE‐Q). Athletes were categorised as recreational (*n* = 72), national (*n* = 94), or elite (*n* = 23), while non‐athlete females were sedentary (*n* = 111) or physically active but non‐competitive (exercisers: *n* = 265). A global EDE‐Q score of > 2.5 was considered as increased risk of an eating disorder. Data were modelled using linear or logistic regression, adjusted for body mass index (BMI), age category, and education level. Global EDE‐Q score was lower among recreational (mean [95% confidence interval]): (1.73 [1.31, 2.14]; *p* = 0.003; *d* = 0.50) and national‐level athletes (1.89 [1.52,2.26]; *p* = 0.024; *d =* 0.39) compared to exercisers (2.47 [2.19,2.75]), with recreational athletes also scoring lower than sedentary females (2.43 [2.09,2.78]; *p* = 0.022; *d* = 0.47). Leanness focused sports had higher restraint (*p* = 0.046; *d =* 0.30) and eating concern (*p* = 0.025; *d* = 0.35) subscale scores compared to non‐leanness focused sports. Recreational‐ or national‐level athletes scored on average lower DEB symptoms using EDE‐Q, compared to sedentary and physical‐active non‐athletes. No EDE‐Q difference was found between competition levels. Leanness focused sport athletes were more concerned about eating behaviours and had higher restraint than athletes from non‐leanness focused sports. These findings suggest that sport participation may be associated with lower or higher disordered eating symptoms, depending on competitive level and type of sport, highlighting the complexity of these relationships in physically active females.

## Highlights

- A high proportion (39.3%) of Norwegian females reported symptoms of disordered eating behaviours. Athletes in leanness focused sports were more likely to report a higher concern around eating behaviours and restraint, compared to non‐leanness focused sports.
- Female athletes competing at recreational‐ or national‐level sport exhibited lower symptoms of disordered eating behaviours compared to physically active non‐athletes.
- Further investigation into the factors and reasons behind the increasing prevalence of disordered eating behaviours among Western females is necessary.

## 1 Introduction

Human eating behaviours span a spectrum, ranging from healthy eating practices to disordered eating behaviours (DEBs), the most severe of which are clinically diagnosed eating disorders (EDs). DEBs encompass various combinations of sub‐optimal or excessive nutritional intake, pathogenic weight control behaviour, and/or increased intrusive thoughts about food, eating, and the body (Reardon et al. 2019). Although symptoms of DEBs in a population may not meet the clinical cut‐offs required for an ED diagnosis, they are recognised as a substantial risk factor and ‘stepping stone’ towards the future development of an ED (Bratland‐Sanda and Sundgot‐Borgen 2013; Klein and Walsh 2004). For instance, different aspects of eating behaviours are associated with DEBs, such as weight concern or purging, have the potential to escalate in severity to an ED, and thus early identification of DEBs may facilitate timely interventions and potentially prevent progression to clinical EDs (Bar et al. 2016). Given the complexity of EDs, which are influenced by a variety of different sociocultural, biological, genetic, and psychological factors (Barakat et al. 2023), the identification of relevant risk factors and DEB symptoms in different populations groups is vital for mitigating the development of clinical EDs.

Athletes may be at an increased risk of developing DEBs compared to non‐athlete populations, particularly elite athletes and/or those competing in certain weight‐sensitive sports (Bratland‐Sanda and Sundgot‐Borgen 2013; Sundgot‐Borgen and Torstveit 2004). This susceptibility may be attributed to various factors specific to athletes, such as an increase in athletic performance associated with lower body mass relative to strength or power for gravity, aesthetics, or weight class sports, such as road cycling, gymnastics, or kickboxing, respectively (Bratland‐Sanda and Sundgot‐Borgen 2013). Such sports are referred to as ‘leanness focused’ due to their emphasis on lower body weight, and athletes of these sports are at higher risk of developing DEBs compared to non‐leanness athletes or non‐athletes (Mancine et al. 2020). In addition to sport‐specific pressures, individual characteristics of athletes may also contribute to risk. For example, the personality traits of successful athletes, such as perfectionism and goal orientation, have also been associated with higher risk of DEB development (Byrne and McLean 2001). What may begin as intentional, healthy dietary practices for athletic performance optimisation may gradually evolve into extreme weight control measures such as self‐induced vomiting or excessive training without adequate nutrition intake (Sundgot‐Borgen and Torstveit 2010).

Previous research has noted that females are at a higher risk of developing DEBs and EDs compared to males, potentially due to differences in social pressures and cultural values (Klein and Walsh 2004; Torres‐McGehee et al. 2023). Female athletes may face increased risk due to their sex and athletic status, when compared to their male athletic counterparts and non‐athletic females (Martinsen et al. 2010; Martinsen and Sundgot‐Borgen 2013; McDonald et al. 2020; Sundgot‐Borgen and Torstveit 2004; Torres‐McGehee et al. 2023). However, a recent meta‐analysis by Chapa et al. (2022) reported similar levels of ED psychopathology between female athletes and non‐athletes, a similar finding as reported earlier (Coelho et al. 2010; Smolak et al. 2000), although heterogeneity between the included studies in these reviews complicates interpretation. Additional factors can also potentially influence DEB risk. For example, regular physical activity may induce a protective effect stemming from enhanced body satisfaction and self‐esteem, possibly offsetting some of the aforementioned DEB risk factors associated with competitive athletes (Bar et al. 2016; Chapa et al. 2022). Thus, additional research is necessary to investigate the prevalence of DEB among female athletes and non‐athletes of different athletic competition and physical activity levels.

Therefore, the objective of this study was to report and compare the prevalence of DEBs among Norwegian female athletes of different competition levels against non‐competitive exercisers and sedentary females, using a validated questionnaire intended to identified DEB symptoms. It was hypothesised that a higher athletic competition level would be associated with increased prevalence of DEBs, whilst physically active females not engaging in sport competitions would have a lower prevalence. Additionally, it was hypothesised that athletes competing in sports emphasising leanness would exhibit a higher prevalence of DEBs compared to athletes competing in sports with lower emphasis on leanness.

## 2 Materials and Methods

### 2.1 Study Design and Recruitment

From September 2022 to March 2023, females residing in Norway and aged 17–40 years were invited to participate in a comparative, cross‐sectional online questionnaire investigating eating behaviours. Recruitment was undertaken via social media posts and word‐of‐mouth. To ensure that the total sample included a sufficient proportion of competing athletes, targeted recruitment via electronic invitations and study information were also sent to coaches, teachers, and support staff at Norwegian sports associations and sports clubs. Prospective participants received comprehensive information regarding the study's objectives and methodology before they provided informed electronic consent, with the understanding that participation in the study was voluntary. As all responses were completely anonymous, both the Regional Ethics Committee for Medical and Health Research and the Norwegian Centre for Research Data confirmed that ethical clearance was not required for this study.

### 2.2 Questionnaire

Participants were asked to complete a customised anonymous online questionnaire, hosted on the survey website Nettskjema (www.nettskjema.no). The questionnaire consisted of an initial demographic information section followed by the Norwegian‐translated self‐report Eating Disorder Examination Questionnaire version 6 (EDE‐Q) (Fairburn and Beglin 1994; Rø et al. 2015). The EDE‐Q, derived from the gold‐standard Eating Disorder Examination Interview, is a widely used questionnaire tool for screening symptoms of EDs (Fairburn and Beglin 1994). To minimise the occurrence of missing answers in the online survey, compulsory responses were implemented throughout the survey, thus requiring participants to complete all closed answer options.

For descriptive purposes, participants reported demographic information such as: their age group (17–20; 21–25; 26–30; 31–35; 36–40 years), height (cm), weight (kg), highest completed education level (primary school; highs school; < 4 years of university; ≥ 4 years of university), county, and average weekly exercise volume (hours) over the preceding 6 months. Body mass index (BMI; kg·m^−2^) was calculated based on the reported height and weight.

Participants were also asked to report their current physical and mental health status, using a seven‐point scale (from 0 ‘*very bad*’ to 6 ‘*very good*’). In addition, participants provided general descriptive information about reproductive factors, such as: pregnancy, current hormonal contraception use and type, average menstrual cycle length (days), presence of oligomenorrhea (menstrual cycle length > 35 days (Taim et al. 2023)), and the number of absent menses over the past 3–4 months. These variables (i.e., self‐rated physical health, self‐rated mental health, and reproductive factors) were collected to provide a broader descriptive health context of the sample but were not central to the primary hypotheses.

The second section of the questionnaire collected eating behaviour data using the Norwegian translated EDE‐Q. The EDE‐Q comprised 28 items, with 22 items categorised into four subscales: *Restraint* (5 items), *Eating Concern* (5 items), *Weight Concern* (5 items), and *Shape Concern* (8 items) (Fairburn and Beglin 1994). The remaining six items assessed the frequency of overeating, binge eating, and compensatory behaviours (e.g., self‐induced vomiting, laxative misuse, and compulsive excessive exercise). Each of the four subscale items were scored from 0 to 6, with higher scores indicating more problematic eating behaviours. The mean of the four subscales provided an overall global EDE‐Q score. A global score of > 2.5 was considered an indication of possible EDs or at‐risk for EDs (i.e., psychopathology), based on previously validated data from the Norwegian population (Rø et al. 2015). In the present study, global EDE‐Q scores were analysed both as a continuous variable (primary outcome) and dichotomised using the > 2.5 cut‐off to classify participants at potential clinical risk (Rø et al. 2015). The Norwegian translation of the EDE‐Q has previously shown satisfactory internal consistency with the English version (Cronbach's *α* = 0.94 for global score; *α* = 0.75–0.90 for the subscales) (Fairburn and Beglin 1994; Rø et al. 2010). It also exhibited robust validity when assessing EDs among Norwegian female university students and strong convergent validity to the original EDE semi‐structured interview (Reas et al. 2011; Rø et al. 2010). Further, it has been validated in recreational to elite‐level athletes, with Cronbach's *α* of 0.805–0.910 for the subscales (Lichtenstein et al. 2021).

### 2.3 Data Cleaning

**FIGURE 1:**
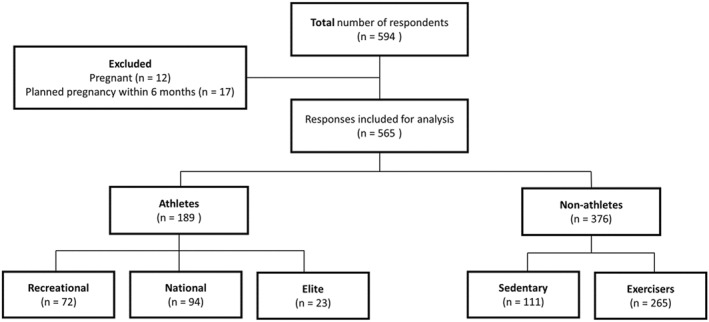
Flow chart of participant inclusion and categorisation from a Norwegian population.

Upon completion of data collection (March 2023) the anonymised dataset was visually inspected for data commonality. A total of 594 females participated in the survey; however, 29 participants were post‐hoc excluded due to either a current pregnancy, or a planned pregnancy within the forthcoming 6 months. A flowchart of participant inclusion and exclusion can be seen in Figure 1. Participants categorisation was developed from the criteria proposed by McKinney et al. (2019) for defining athletes and exercisers, namely that the intent of the physical activity distinguished the two groups. As such, participants were considered as athletes if they currently participated in competitive sport, or non‐athletes if they were not participating in competitive sport. Non‐athletes were classified as either *sedentary* (< 2.5 h exercise·week^−1^) or *exerciser* (≥ 2.5 h exercise·week^−1^), while the athletes were sub‐divided into three groups based on their current sporting competitive level: *recreational* (regional and local level); *national; elite* (international level) (McKinney et al. 2019). Athletes' primary sport was also used to categorise them into two distinct categories, leanness focused (i.e., aesthetic, endurance, and weight‐class sports) or non‐leanness focused (i.e., ball, power, and technical sports), adapted from previous work (Mancine et al. 2020; Martinsen et al. 2010; Torstveit and Sundgot‐Borgen 2005). See Table 1 for athlete sport classification into leanness focused or non‐leanness focused. The seven‐point scale variables of self‐evaluated mental health and self‐evaluated physical health were treated as continuous data.

**TABLE 1:** Classification of athletes' (*n* = 189) primary sport into leanness focused or non‐leanness focussed categories.

Leanness focused sports (*n* = 95)			Non‐leanness focused sports (*n* = 94)		
Aesthetic (*n* = 5)	Endurance (*n* = 75)	Weight‐class (*n* = 15)	Ball (*n* = 89)	Power (*n* = 2)	Technical (*n* = 3)
Diving (*n* = 3) Cheerleading (*n* = 1) Rhythmic gymnastics (*n* = 1)	Swimming (*n* = 57) Triathlon (*n* = 3) Cross‐country skiing (*n* = 6) Running (*n* = 8) Long‐track speed skating (1)	Taekwondo (*n* = 3) Karate (*n* = 3) Brazilian jiu jitsu (*n* = 2) Olympic weightlifting (*n* = 3) Powerlifting (*n* = 4)	Ice hockey (*n* = 13) Football (*n* = 10) Handball (*n* = 50) Floorball (*n* = 5) Volleyball (*n* = 3) Lacrosse (*n* = 2) Badminton (*n* = 5) Basketball (*n* = 1)	Short distance running (*n* = 1) Short‐track speed skating (*n* = 1)	Dressage (*n* = 1) Horseback riding (*n* = 1) Sailing (*n* = 1)

The EDE‐Q data followed the published scoring guide, with four distinct subscales (restraint, eating concern, weight concern, and shape concern) and an overall mean score (global score) (Fairburn 2008). Each subscale required a majority (> 50%) response rate to be considered valid. As part of the EDE‐Q, frequency of overeating, binge eating, and compensatory behaviours over the preceding 28 days were collected (EDE‐Q questions 13–18 (Fairburn and Beglin 1994)). Overeating episodes were distinguished as the consumption of a large amount of food (considered by others as a large quantity of food, given the circumstances) and categorised as objective overeating (not loss of control) or objective binge eating (self‐perceived loss of control). Regular occurrence of overeating, binge eating and compensatory behaviour episodes over the preceding 28 days was defined as: ≥ 5 episodes of compulsive excessive exercise per week, and ≥ 4 occurrences of laxative misuse, self‐induced vomiting, binge eating, or overeating (i.e., at least once per week) over the preceding 28 days (Rø et al. 2010).

### 2.4 Statistical Analysis

All data analyses were undertaken using R in the RStudio environment (R Core Team 2020).

An a priori power analysis was undertaken using the package ‘pwr’ (Champely et al. 2020). Data from a similar study comparing global EDE‐Q scores of female university students across different physical activity and competition levels (Darcy et al. 2013) was used to determine the minimum sample size necessary to compare five groups: *n* = 67 per group (80% power; *α* = 0.05; 4 coefficients).

### 2.5 Demographic Differences

Differences in demographic variables between the factor of ‘*group*’ (levels: sedentary; exercisers; recreational athletes; national athletes; elite athletes) were analysed using linear regression (height; weight; BMI; self‐evaluated mental health; self‐evaluated physical health), binominal logistic regression (hormonal contraceptive use; oligomenorrhea), multinominal regression (type of hormonal contraceptive) or cumulative‐link ordinal regression (average menstrual cycle length; age category; highest education level) (Christensen 2022).

### 2.6 Association Between Group and EDE‐Q Data

The association between the fixed factor *group* and the continuous score from the EDE‐Q (i.e., global score and each of the four subscales) was modelled with linear regression. Binomial logistic regression was used to model the association between *group* and the binary dependent variable of clinical EDs (i.e., EDE‐Q global score > 2.5), while negative binomial regression was used to model the association between *group* and overdispersed occurrence data of overeating, binge eating and compensatory behaviours via the ‘MASS’ package (Ripley et al. 2024). The association between *sport category* (levels: leanness; non‐leanness) and the EDE‐Q global score were also modelled with linear regression. All models included adjustments for potential confounders of *BMI*, *age category*, and *highest education level,* given their possible association with DEB risk (Carrard and Rothen 2020; Fitzsimmons‐Craft et al. 2019; Romano et al. 2020; Tabler and Utz 2015). Models were tested for excessive multicollinearity (variance inflation factor > 10) value using the ‘performance’ package (Lüdecke et al. 2021) to produce variance inflation factors.

Model fit, dispersion, multicollinearity, and convergence of all models were checked using the ‘performance’ package (Lüdecke et al. 2021). Post‐hoc testing, effect sizes (e.g., Cohen's *d*), and adjusted means were produced using the ‘emmeans’ package (Lenth et al. 2020), with multivariate *t*‐adjustment. Statistical significance was assumed at *α* = 5%. Descriptive statistics of the study participants are presented in frequency counts and percentages, whilst other data are provided as (adjusted) means and 95% confidence intervals (95% CI), or Odds Ratio (OR) with 95% CI, unless otherwise noted.

## 3 Results

A total of 565 respondents were included in the analyses: 189 athletes and 376 non‐athletes. Respondents were located across all regions of Norway: north (*n* = 67); middle (*n* = 77); west (*n* = 180); south (*n* = 20); and, east (*n* = 221). See Table 2 for demographic data of the total sample and for each group, and Table S1 provides additional demographic information (e.g., reproductive factors). The athlete group were also categorised as either leanness focused sports (*n* = 95) or non‐leanness focused sports (*n* = 94).

**TABLE 2:** Demographic and anthropometric characteristics of female athletes and non‐athletes in Norway.

Variable	All (*n* = 565)	Non‐athletes (*n* = 376)		Athletes (*n* = 189)		
		Sedentary (*n* = 111)	Exercisers (*n* = 265)	Recreational (*n* = 72)	National (*n* = 94)	Elite (*n* = 23)
Body mass (kg)	68.8 ± 15.0	73.7 ± 19.5	68.1 ± 15.1	67.5 ± 11.4	66.9 ± 10.6	65.0 ± 9.3
Height (cm)	168 ± 7	169 ± 6	168 ± 7	169 ± 7	168 ± 7	170 ± 7
BMI (kg/m^−2^)	24.2 ± 4.8	25.8 ± 6.2	24.0 ± 4.9	24.1 ± 3.9	23.3 ± 3.3	22.7 ± 2.3
Self‐evaluated physical health (1 = very bad to 6 = very good)	3.6 ± 1.3	4.0 ± 1.1^a^	3.0 ± 1.1	4.2 ± 1.1^a^	4.5 ± 1.1^a^	4.2 ± 1.0^a^
Self‐evaluated mental health (1 = very bad to 6 = very good)	3.3 ± 1.4	3.3 ± 1.3	3.0 ± 1.4	3.8 ± 1.3^b^	3.5 ± 1.2	3.7 ± 1.6
Training history (years)	—	—	—	9.8 ± 4.8	9.8 ± 4.7	10.8 ± 6.3
Age categories (years)						
17–20	156 (27.6%)	14 (12.6%)	43 (16.2%)	34 (47.2%)	53 (56.4%)	12 (52.2%)
21–25	174 (30.8%)	40 (36.0%)	89 (33.6%)	18 (25.0%)	19 (20.2%)	8 (34.8%)
26–30	155 (27.4%)	33 (29.7%)	92 (34.7%)	14 (19.4%)	14 (14.9%)	2 (8.7%)
31–35	70 (12.4%)	20 (18.0%)	38 (14.3%)	6 (8.3%)	6 (6.4%)	0 (0.0%)
36–40	10 (1.8%)	4 (3.6%)	3 (1.1%)	0 (0.0%)	2 (2.1%)	1 (4.3%)

*Note:* Data presented as unadjusted means ± standard deviation, or frequency (valid % of group).

Abbreviations: BMI, body mass index.

^a^ Significantly different to *exercisers* group.

^b^ Significantly different to *sedentary* group.

### 3.1 Global EDE‐Q Score Differences Between Female Athletes and Non‐Athlete Groups

**FIGURE 2:**
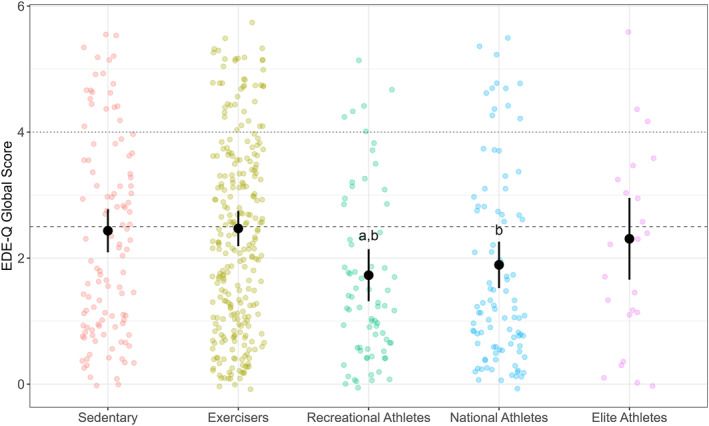
Eating Disorder Examination Questionnaire (EDE‐Q) global scores for females who are either sedentary, not engaged in competitive sport but exercise, or participate in competitive sport at different levels (recreational; national; elite) in a Norwegian population. Data presented as raw participant scores (coloured points), adjusted group means indicated with a solid black point, and black vertical lines indicate 95% confidence intervals. Means are adjusted for BMI, age, and highest education level. Horizontal line represents the clinical threshold cut‐offs for possible ED psychopathology, based on reference data for Norwegian females (> 2.5 threshold; Rø et al. 2015; dashed line), and the original EDE‐Q (> 4.0 threshold; dotted line). Group differences are indicated as: ^a^ = significantly lower than *sedentary* group; ^b^ = significantly lower than *exercisers* group.

There were significant associations between *group* and global EDE‐Q score (*p* = 0.001). Post‐hoc analysis found that recreational athletes scored significantly lower on the EDE‐Q global score (mean: 1.73 [95% CI: 1.31, 2.14]) compared to sedentary (2.43 [2.09, 2.78]; *p* = 0.022; *d* = 0.47 [0.16, 0.78]) while both recreational (*p* = 0.003; *d* = 0.50 [0.22, 0.77]) and national level athletes (1.89 [1.52, 2.26]; *p* = 0.024; *d* = 0.39 [0.13, 0.65]) had lower global scores than exercisers (2.47 [2.19, 2.75]; Figure 2). A statistical difference was also found between *group* and clinical ED occurrence (*p* = 0.011), with exercisers scoring within a higher likelihood of clinical EDs compared to recreational athletes (*p* = 0.012; OR = 2.83 [1.17, 6.86]; Table 3). The global EDE‐Q score of the elite athlete group (2.31 [1.66, 2.96]), as well as their likelihood of scoring in the clinical ED range, was not significantly different from any other group.

**TABLE 3:** Proportion of female athletes and non‐athletes engaging in overeating, binge eating, and compensatory behaviours using the Eating Disorder Examination Questionnaire (EDE‐Q) in Norway.

		All (*n* = 565)	Non‐athletes (*n* = 376)		Athletes (*n* = 189)		
			Sedentary (*n* = 111)	Exercisers (*n* = 265)	Recreational (*n* = 72)	National (*n* = 94)	Elite (*n* = 23)
Global EDE‐Q score > 2.5		39.3% (*n* = 222)	44.1% (*n* = 49)	45.3% (*n* = 120)	22.2%^a^ (*n* = 16)	29.8% (*n* = 28)	39.1% (*n* = 9)
Eating and compensatory behaviours	Occurrence in past 4 weeks						
Objective overeating (episodes)	Any	46.3%	44.0%	48.5%	49.3%	43.5%	34.8%
	Regular (≥ 1/week)	29.3%	29.4%	29.2%	36.2%	27.2%	17.4%
Objective binge eating (episodes)	Any	33.2%	35.2%	39.1%	32.4%	18.5%	17.4%
	Regular (≥ 1/week)	18.8%	20.4%	21.8%	22.1%	8.7%	8.7%
Self‐induced vomiting (episodes)	Any	7.3%	6.3%	9.1%	5.6%	5.3%	4.3%
	Regular (≥ 1/week)	3.4%	3.6%	3.8%	2.8%	3.2%	0.0%
Laxative misuse (episodes)	Any	2.8%	1.8%	2.3%	1.4%	5.3%	8.7%
	Regular (≥ 1/week)	2.3%	0.9%	1.9%	1.4%	4.3%	8.7%
Excessive exercise (episodes)	Any	46.8%	30.0%	56.8%	52.1%	37.6%	36.3%
	Regular (≥ 5/week)	11.8%	3.6%	14.5%	13.0%	12.9%	13.6%

*Note:* Data presented as valid proportion of group, calculated from participants with valid (non‐missing) responses for each specific behaviour. Denominators may therefore vary between behaviours. A global EDE‐Q score > 2.5 indicates potential clinical eating disorder psychopathology, based on reference data for Norwegian females (Rø et al. 2015).

^a^ Group differences are indicated as significantly lower than *exercisers* group.

**FIGURE 3:**
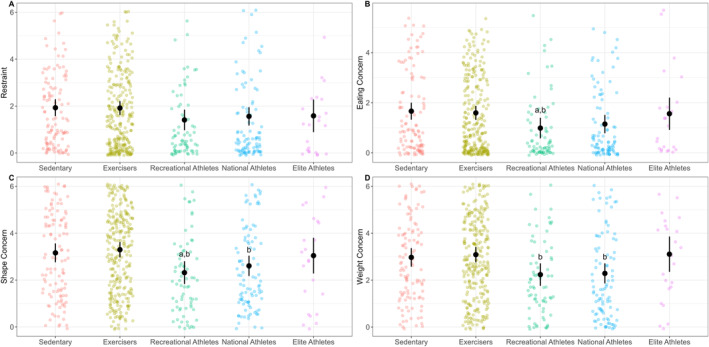
Eating Disorder Examination Questionnaire (EDE‐Q) subscale scores for females who are either sedentary, not engaged in competitive sport but exercise, or participate in competitive sport at different levels (recreational; national; elite) in Norway. Subscales are: (A) restraint, (B) eating concern, (C) shape concern, and (D) weight concern. Data presented as raw participant scores (coloured points), adjusted group means indicated with a solid black point, and black vertical lines indicate 95% confidence intervals. Means are adjusted for BMI, age, and highest education level. Group differences are indicated as: ^a^ = significantly lower than *sedentary* group; ^b^ = significantly lower than *exercisers* group.

A main effect for *group* was found for the subscales of eating concern (*p* = 0.011), shape concern (*p* < 0.001), and weight concern (*p* < 0.001). No significant main effect of *group* was found for the restraint subscale (*p* = 0.069). Post‐hoc analysis for the subscales found that recreational athletes (0.99 [0.58, 1.40]) had significantly lower eating concern compared to exercisers (1.60 [1.32, 1.87]; *p* = 0.026; *d* = 0.41 [0.14, 0.68]) and the sedentary group (1.66 [1.32, 2.00]; *p* = 0.030; *d* = 0.46 [0.16, 0.76]; Figure 3). Both recreational (2.32 [1.83, 2.80]) and national (2.61 [2.17, 3.04]) athlete groups also scored significantly lower on shape concern, compared to exercisers (3.30 [2.97, 3.63]; *p* = 0.001 to 0.021; *d* = 0.39 [0.14, 0.65] to 0.56 [0.28 to 0.83]), and recreational athletes also scored lower than the sedentary group (3.16 [2.76, 3.56]; *p* = 0.018; *d* = 0.48 [0.16, 0.79]; Figure 3). Weight concern was significantly lower for recreational (2.24 [1.76, 2.72]) and national (2.29 [1.86, 2.71]) athletes compared to exercisers (3.09 [2.35, 3.86]; *p* = 0.003 to 0.004; *d* = 0.46 [0.20 to 0.72] to 0.49 [0.22 to 0.77]; Figure 3). No differences were found for elite athletes on any subscale.

### 3.2 Group Differences for Overeating, Binge Eating and Compensatory Behaviours

No significant differences were found between the fixed factor of *group* and the frequency of occurrence of overeating (*p* = 0.898), binge eating (*p* = 0.290) or any of the compensatory behaviours, such as self‐induced vomiting (*p* = 0.354), laxative misuse (*p* = 0.648) or excessive exercise (*p* = 0.060). The prevalence of these behaviours for each group are provided in Table 3.

### 3.3 Sport Category (Leanness Focused vs. Non‐Leanness Focused) Differences for Athletes in EDE‐Q Data

**FIGURE 4:**
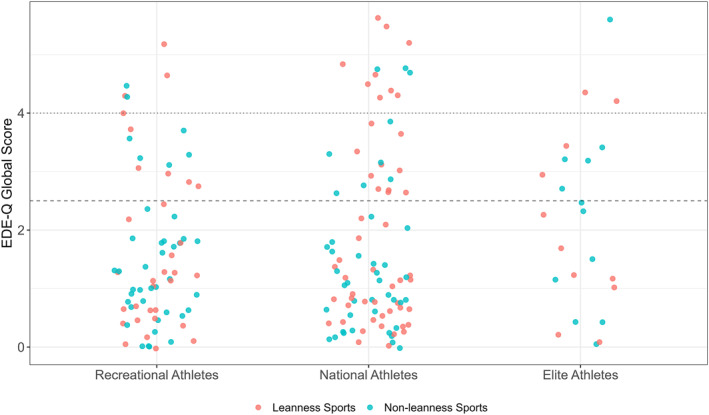
Eating Disorder Examination Questionnaire (EDE‐Q) global scores for female athletes in Norway with the primary sport categorised as leanness focused or non‐leanness focused. Data presented as raw participant scores, with the point colour indicating the participant's sport categorisation: red = leanness focused sports; blue = non‐leanness focused sports. Horizontal lines represent the clinical threshold cut‐offs for possible ED psychopathology, based on reference data for Norwegian females (> 2.5 threshold; Rø et al. 2015; dashed line), and the original EDE‐Q (> 4.0 threshold; dotted line).

**FIGURE 5:**
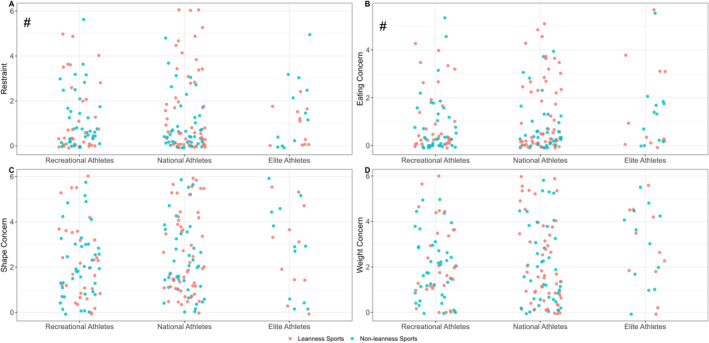
Distribution of Eating Disorder Examination Questionnaire (EDE‐Q) subscale scores for female athletes in Norway with a primary sport that is categorised as leanness focused or non‐leanness focused. Data presented as raw participant subscale scores, with the point colour indicating the participant's sport categorisation: red = leanness focused sports; blue = non‐leanness focused sports. Subscales are: (A) restraint, (B) eating concern, (C) shape concern, and (D) weight concern. Significant subscale score differences (i.e., *p* < 0.05) between leanness focused and non‐leanness focused sports, independent of competition level, are indicated by #. See Table 2 for the classification of sports into these two separate categories.

No significant difference was found between leanness focused and non‐leanness focused sport athletes for global score (*p* = 0.088). However, leanness focused sport athletes reported significantly higher scores for the subscales of restraint (*p* = 0.046; *d* = 0.30 [0.01, 0.59]) and eating concern (*p* = 0.025; *d* = 0.34 [0.04, 0.64]) compared to athletes competing in non‐leanness focused sports. Global and subscale scores for leanness focused and non‐leanness focused athletes can be seen in Figures 4 and 5, respectively.

## 4 Discussion

The objective of this study was to report and compare the prevalence of DEBs among Norwegian females of different physical activity levels, ranging from sedentary to elite‐level athletes, using the EDE‐Q screening tool to identify symptoms. The primary finding of this study was that the prevalence of DEBs was high across the total sample, with 39.3% of participants scoring above the clinical cut‐off on the global EDE‐Q. Furthermore, athletes competing at a recreational‐ or national‐level scored lower on the global EDE‐Q score when compared to exercising non‐athletes. Similarly, recreational‐ and national‐level athletes reported lower concern for their shape and weight, when compared to exercisers. In contrast to the hypothesis that higher‐level athletes would have higher scores of DEBs, no differences in either global or subscale scores of the EDE‐Q were found between different athletic competition levels (Figures 2 and 3). However, consistent with our second hypothesis, athletes participating in leanness focused sports scored higher on the restraint and eating concern sub‐scales compared to those in non‐leanness focused sports, though no differences were observed for shape or weight concern (Figure 5). These data suggests that participation in competitive sport, particularly for females who are already physically active, is associated with a lowered likelihood of DEBs.

It was hypothesised that competitive athletes would report higher global EDE‐Q scores compared to the non‐athlete groups, due to heighted pressures such as sport‐specific appearance ideals, perfectionism, and the association between lower body mass and potentially increased athletic performance for certain sports (Byrne and McLean 2001; Sundgot‐Borgen and Torstveit 2010). Contrary to this hypothesis, the recreationally competitive‐ and national‐level athlete groups recorded lower mean global EDE‐Q scores than the physically active non‐athlete group (i.e., *exercisers*), with the recreational athletes also lower than the sedentary group. Some earlier Norwegian studies reported similar or higher prevalence of possible DEB risk among athletes compared to non‐athletes when classified using various screening tools (Martinsen et al. 2010; Sundgot‐Borgen and Torstveit 2010). However, several systematic reviews have noted that the use of different questionnaires, and therefore, different scoring systems, across studies poses a challenge to directly comparing and summarising DEB prevalence (Chapa et al. 2022; Coelho et al. 2010; Smolak et al. 2000). A more reasonable comparison for the present data is against prior research utilising versions of the EDE‐Q, where no significant differences in global EDE‐Q score were reported between athletes and control groups (Carvalhais et al. 2019; Hulley et al. 2007; Mathisen et al. 2020). Similarly, while competition level has been investigated in a limited number of studies, two UK‐based studies found that competitive athletes exhibited a higher risk of disordered eating behaviours compared to recreational athletes (Dervish et al. 2023; Sharps et al. 2022); however, competition level accounted for only a modest proportion of variance. Furthermore, both studies used different questionnaire tools than the EDE‐Q, limiting direct comparability. Taken together, these findings suggest that competitive level alone is not a strong determinant of DEB risk, and that non‐athletic physically active females (i.e., exercisers) may also be vulnerable to disordered eating behaviours.

The prevalence of participants with DEBs was high in this study, with almost two‐fifths (39.3%; Table 3) of the total sample reporting a global EDE‐Q score that surpassed an ED cut‐off threshold of 2.5 (based on previously validated Norwegian data (Rø et al. 2015)). More specifically, over a quarter (28%) of athletes and 45% of non‐athletes scored above this clinical threshold. These findings are considerably higher than previous research which used a two‐phase approach (questionnaire‐based screening followed by clinical interviews), where only a fifth of athletes and 9% of non‐athletes met the clinical ED criteria (Sundgot‐Borgen and Torstveit 2004). Notably, the study by Sundgot‐Borgen and Torstveit (2004) used the Eating Disorder Inventory 2 (EDI‐2) for initial screening, which includes psychological subscales not assessed by the EDE‐Q, potentially contributing to differences in identification of ED cases. Moreover, the study identified a high rate of false positives, particularly among non‐athletes, when comparing questionnaire results to clinical interviews (Sundgot‐Borgen and Torstveit 2004). Thus, the sole reliance on the EDE‐Q questionnaire in the present study may have led to an overestimated risk of EDs. This overestimation could potentially explain the higher proportion of non‐athletes with potential EDs (i.e., global EDE score > 2.5) observed in the current study compared to previous findings, highlighting the need to interpret the current prevalence data with caution. Alternatively, the observed differences may reflect the considerable societal changes in the two decades since this previous study was conducted (e.g., increased globalisation, access to social media, etc.) (Sundgot‐Borgen and Torstveit 2004), warranting the need for additional research to confirm these findings.

The global EDE‐Q scores of the present study were also high, particularly for the non‐athlete groups, when compared to normative values from other studies using the EDE‐Q. For instance, adjusted mean global EDE‐Q scores for non‐athletes in the present study ranged from 2.43 to 2.47, substantially higher than scores reported for Norwegian females (1.09–1.42; Mathisen et al. 2020; Reas et al. 2011; Rø et al. 2010, 2015), or similar populations in Australia (1.52; Mond et al. 2006), Sweden (1.56; Welch et al. 2011), the USA (1.74; Luce et al. 2008), or the UK (1.82; Hulley et al. 2007). Considering the established validity of the EDE‐Q tool in the Norwegian population (Reas et al. 2011) and the large non‐athlete sample size of this study (*n* = 376), this finding was unexpected. One possible explanation for the proportion of scores above the clinical cut‐off may be the lingering effect of the COVID‐19 pandemic, which has been linked to a decline in overall mental health and increased rates of DEBs (Firoozjah et al. 2022; Linardon et al. 2022). Although initial triggers for DEB occurrence, such as social isolation, routine disruption, and/or heightened stress, may have diminished since the pandemic, the residual effects on mental health and eating behaviours may still persist (Rodgers et al. 2020). This trend might partly explain why the prevalence of DEB in this study is higher than earlier reports, or as previously mentioned, this difference may also reflect a broader increase in such behaviours over time since earlier studies.

Physical activity has been suggested to potentially provide a protective effect against DEBs, reducing body‐image concern and improving self‐esteem (Chapa et al. 2022; Smolak et al. 2000). However, a larger proportion of the physically active non‐athlete group (i.e., *exercisers*) in the present study reported higher subscale scores for shape and weight concern compared to both the proportion of recreational and national‐level athletes and reported more eating concern compared to recreational athletes alone. These findings are consistent with the observations of Darcy et al. (2013), who noted that athletes focussing on competitive sport displayed the lowest levels of ED‐related psychopathology. Similarly, Torstveit et al. (2015) reported that students in sports‐focused programs had the lowest DEB prevalence when compared to students in general‐ or vocational programs. Altogether, these data suggest that it may be the additional competitive aspect that is inherent in organised sport that may lead to lower prevalence of DEBs, as opposed to just physical activity (Chapa et al. 2022). For example, athletes may place a higher emphasis on healthy eating behaviours in order to optimise their athletic performance, such as ensuring adequate nutritional fuelling to complete a football match (Bentley et al. 2019). Athletes have also reported that sports dietitians are a preferred source of dietary information (Trakman et al. 2019), and may have easier access to these professionals through their sporting organisations (Hull et al. 2016), which may be less readily accessible to non‐athletes. In contrast, physically active non‐athletes may obtain their knowledge around nutrition and exercise from lower‐quality sources, such as influencers on social media, and therefore may be exposed to unrealistic body figure expectations and questionable eating behaviours (Dane and Bhatia 2023).

Higher scores for restraint and eating concern subscales were observed for athletes from leanness focused sports, compared to non‐leanness focused sports, although no differences were noted for shape or weight concern. This may be attributed to the sport type distribution within the leanness category, as the majority of athletes represented endurance sports (79%), and thus may have lower emphasis on shape concern when compared to aesthetic sports (Mancine et al. 2020). Future research should specifically investigate if the leanness focussed sport type (e.g., endurance, aesthetic, weight‐class) alters EDE‐Q subscale responses. Although no significant differences were found between leanness focused and non‐leanness focused sports for the global EDE‐Q score in the present study, this may have been due to insufficient power for the comparison, illustrated by a non‐significant difference (*p* = 0.088). When taken together, these data align with previous evidence on Norwegian athletes, namely that sports emphasising leanness often coincide with higher levels of DEBs (Chapa et al. 2022; Sundgot‐Borgen 1994; Sundgot‐Borgen and Torstveit 2004; Torstveit et al. 2008). Interestingly, younger Norwegian athletes (i.e., 15–16 years) do not seem to exhibit differences in DEB prevalence between leanness focused and non‐leanness focused sports, possibly due to the shorter timeframe that they have been involved in sports and so a limited exposure to leanness focused sport specific dietary pressures (Martinsen et al. 2010).

### 4.1 Practical Implications

The high proportion of participants with EDE‐Q global scores above the clinical cut‐off is concerning, and may potentially reflect an increasing prevalence of DEBs among Norwegian females compared to earlier studies. The implementation of comprehensive educational programs about DEBs, particularly focussing on reducing concerns about weight and body shape (e.g., fear of weight gain, influence of body shape on self‐worth), may be beneficial in decreasing DEB prevalence. Additionally, preventive measures such as early screening and intervention strategies should be considered to mitigate the risk of DEBs developing into more serious conditions. Qualitative research has also highlighted the potential power imbalance between athletes and coaches, particularly for female and/or young athletes, as a barrier to seeking help for DEBs (Fatt et al. 2024). Therefore, it is crucial that coaching staff receive education about healthy eating behaviours and the signs of DEBs to create a supportive environment that encourages athletes to seek help. Although the EDE‐Q has been validated for use in athletes (Lichtenstein et al. 2021), the development of athlete‐specific DEB screening and clinical assessment tools to ensure more accurate identification of DEB in this population should be considered.

### 4.2 Limitations and Strengths

Several limitations of this study warrant consideration. Firstly, participants in this group were primarily recruited via social media, which could introduce selection bias towards females who are predominantly active on social media platforms. Exposure to social media, particularly fitness influencers promoting unrealistic body standards and potentially harmful eating practices, has been identified as a risk factor for development of DEBs, particularly among females (Dane and Bhatia 2023). Participants with current EDs were also not excluded from the data collection, potentially skewing the sample. Secondly, the EDE‐Q is a widely‐used self‐report screening tool (Chapa et al. 2022; Coelho et al. 2010), and the Norwegian version has demonstrated good reliability and strong convergent validity to the original EDE semi‐structured interview (Reas et al. 2011; Rø et al. 2010). However, such self‐report questionnaires may result in false‐positives, potentially overestimating ED prevalence (Sundgot‐Borgen and Torstveit 2004). Finally, an underrepresentation of elite athletes in our study population (*n* = 23) may have introduced bias into the results, as this group was likely underpowered based on the a priori calculated minimum group sample size (group *n* = 67). Therefore, while the present findings suggest no discernible difference in DEB prevalence across competition levels, these data warrant careful interpretation.

Despite the acknowledged limitations, the present study also exhibits several strengths. The use of anonymous self‐report data may have mitigated a social desirability bias regarding the potentially sensitive topic of EDs, improving data accuracy. Additionally, athletes may have felt more at ease providing truthful responses in an anonymous format, knowing that their athletic career would not be jeopardised regardless of the answers provided, thus reducing the likelihood of underreporting (Sundgot‐Borgen 1993). The use of an online survey also resulted in the inclusion of participants from across all regions of Norway rather than a specific location, strengthening the generalisability of the results across Norway.

## 5 Conclusion

In this cross‐sectional study among Norwegian athletes and non‐athletes, the prevalence of DEBs was high across the total sample. However, both recreational‐ and national‐level athlete groups scored lower on the global EDE‐Q when compared to exercising non‐athletes, with recreational athletes also scoring lower than sedentary females. These findings add to existing evidence of the burden of DEBs among females across different levels of physical activity. Future investigations are needed to confirm the current findings and to better understand factors contributing to the presence of DEBs among females, both within athletic and general populations.

## Author Contributions

M.M.S.V., E.H.S., K.B.B., and J.O.O. conceived and designed the study. M.M.S.V. collected and cleaned the data. J.O.O. and M.M.S.V. undertook the data analyses and wrote the first draft. All authors (M.M.S.V., E.H.S., K.B.B., M.K.T., and J.O.O.) were substantially involved in the data interpretation and critical revision of the manuscript throughout the drafting process, as well as approving the final version of the manuscript for submission.

## Conflicts of Interest

The authors declare no conflicts of interest.

## Supporting information


**Table S1**: Demographic and anthropometric characteristics of female athletes and non‐athletes in Norway.

## Data Availability

The data that support the findings of this study are available from the corresponding author upon reasonable request.
