# Supplementary material for: Lower Prevalence of Disordered Eating Behaviours Among Norwegian Female Athletes Compared to Non‐Athletes: A Cross‐Sectional Survey Using the Eating Disorder Examination Questionnaire
Source: Eur J Sport Sci. 2025 Aug 20;25(9):e70043. doi: 10.1002/ejsc.70043 (PMC12365784; doi:10.1002/ejsc.70043)
Supplement: Supplementary file 1 — Table S1: Demographic and anthropometric characteristics of female athletes and non‐athletes in Norway. [file EJSC-25-e70043-s001.docx]

**Supplementary Table 1. Demographic and anthropometric characteristics of female athletes and non-athletes in Norway.**

| Variable | All (n = 565) | | Non-Athletes (n = 376) | | | Athletes (n = 189) | | | |  |
| --- | --- | --- | --- | --- | --- | --- | --- | --- | --- | --- |
|  |  |  | Sedentary  (n = 111) | Exercisers  (n = 265) | | Recreational  (n = 72) | National  (n = 94) | | Elite  (n = 23) |  |
| Menstrual cycle length | |  | | |  | | |  | | |
| <20 days | 27 (4.8%) | | 5 (4.5%) | 10 (10.8%) | | 6 (8.3%) | 6 (6.4%) | | 0 (0.0%) |  |
| 21-34 days | 422 (74.7%) | | 77 (69.4%) | 204 (77.0%) | | 54 (75.0%) | 69 (73.4%) | | 18 (78.3%) |  |
| >35 days | 116 (20.5%) | | 29 (26.1%) | 51 (19.2%) | | 12 (16.7%) | 19 (20.1%) | | 5 (21.7%) |  |
| Hormonal contraceptive use | 344 (60.9%) | | 70 (63.1%) | 161 (60.8%) | | 43 (59.7%) | 54 (57.4%) | | 16 (69.6%) |  |
| Hormonal contraceptive type |  | |  |  | |  |  | |  |  |
| Intrauterine system (IUS) | 95 (27.6%) | | 18 (25.7%) | 54 (33.5%) | | 8 (18.6%) | 10 (18.5%) | | 5 (21.7%) |  |
| Hormonal implant | 69 (20.1%) | | 12 (17.1%) | 23 (14.3%) | | 14 (32.6%) | 15 (27.8%) | | 5 (21.7%) |  |
| Combined oral contraceptives | 140 (40.7%) | | 28 (40.0%) | 65 (40.4%) | | 17 (39.5%) | 26 (48.1%) | | 4 (25.0%) |  |
| Progestin-only oral contraceptives | 28 (8.1%) | | 8 (11.4%) | 13 (8.1%) | | 4 (9.3%) | 1 (1.9%) | | 2 (12.5%) |  |
| Other (injection, patch, vaginal ring) | 12 (3.5%) | | 4 (5.7%) | 6 (3.7%) | | 0 (0.0%) | 2 (3.7%) | | 0 (0.0%) |  |

Data presented as frequency (valid % of group). Type of hormonal contraceptive percentage is calculated only for hormonal contraceptive users of that group.
